# Evaluation of Specific Symptoms of Bacterial Vaginosis Among Pregnant Women

**DOI:** 10.1155/S1064744997000653

**Published:** 1997

**Authors:** J. Boris, T. B. Henriksen, U. Davidsen, N. J. Secher

**Affiliations:** 1 Department of Obstetrics and Gynecology Hospital of Skaraborg Skövde Skövde S-541 85 Sweden; 2 Perinatal Epidemiological Research Unit Aarhus University Hospital Aarhus Denmark

## Abstract

*Objective:* Identification of the symptoms of bacterial vaginosis (BV) in pregnancy might be rational in order to identify a possible BV-associated group at risk of preterm delivery.

*Methods:* Three hundred and five women early in the third trimester of pregnancy were interviewed about lifestyle factors and specific symptoms of BV and given a vaginal examination. A longitudinal three-week follow-up was conducted for 127 women.

*Results:* The prevalence of BV was 16%. Women with BV were significantly more often smokers than women without BV (52% vs. 34%). No difference in sexual activity or other behavioral characteristics between the two groups were seen. No differences were noted among women with and without BV according to specific symptoms: malodorous discharge (26% vs. 23%), increased discharge (76% vs. 68%), or itching or troublesome discharge. More than one third of women with BV at the first examination did not fulfill the criteria for BV at the three week follow-up exam. None of the women without BV had developed BV by the follow-up exam. The incidence of preterm delivery among women with BV was 4%, women without BV had an incidence of 2.4%. This difference was not statistically significant.

*Conclusion:* Asymptomatic BV in pregnancy is common. Specific questions about the character of the discharge do not identify women with BV during pregnancy. To identify a potential BV-associated group at risk for preterm delivery, screening for BV must be conducted not only among symptomatic women but among all women. Women with BV are more often smokers than women without BV.


Infectious Diseases in Obstetrics and Gynecology 5:361–365 (1997)
© 1998 Wiley-Liss, Inc.

### Evaluation of Specific Symptoms of Bacterial Vaginosis Among Pregnant Women

J. Boris,[1]* T.B. Henriksen,[2] U. Davidsen,[2] and N.J. Secher[2]

[1]*Department of Obstetrics and Gynecology, Hospital of Skaraborg, Skövde, Sweden*

[2]*Perinatal Epidemiological Research Unit, Aarhus University Hospital, Aarhus, Denmark*

### Abstract

*Objective:* Identification of the symptoms of bacterial vaginosis (BV) in pregnancy might be rational in order to identify a possible BV-associated group at risk of preterm delivery.

*Methods:* Three hundred and five women early in the third trimester of pregnancy were interviewed about lifestyle factors and specific symptoms of BV and given a vaginal examination. A longitudinal three-week follow-up was conducted for 127 women.

*Results:* The prevalence of BV was 16%. Women with BV were significantly more often smokers than women without BV (52% vs. 34%). No difference in sexual activity or other behavioral characteristics between the two groups were seen. No differences were noted among women with and without BV according to specific symptoms: malodorous discharge (26% vs. 23%), increased discharge (76% vs. 68%), or itching or troublesome discharge. More than one third of women with BV at the first examination did not fulfill the criteria for BV at the three week follow-up exam. None of the women without BV had developed BV by the follow-up exam. The incidence of preterm delivery among women with BV was 4%, women without BV had an incidence of 2.4%. This difference was not statistically significant.

*Conclusion:* Asymptomatic BV in pregnancy is common. Specific questions about the character of the discharge do not identify women with BV during pregnancy. To identify a potential BV-associated group at risk for preterm delivery, screening for BV must be conducted not only among symptomatic women but among all women. Women with BV are more often smokers than women without BV. Infect. Dis. Obstet. Gynecol. 5:361–365, 1997. © 1998 Wiley-Liss, Inc.

Key words bacterial vaginosis; premature labor; pregnancy; smoking; symptoms

Bacterial vaginosis (BV) has been linked to numerous gynecologic and obstetric complications. Bacterial vaginosis in pregnancy has been associated with increased risk of preterm delivery and delivery of low-birth-weight infants[1–4] and postpartum complications, such as endometritis and postcesarean infections.[5]

The most critical period in pregnancy concerning vaginal infections seems to be the first and second trimesters.[6–8] The presence of an abnormal bacterial flora in early and mid pregnancy is thought to induce labor by stimulating the production of bacterial cytolytic enzymes and substances produced by bacteria-induced inflammatory cells or result from a subclinical infection in the uterus.[6]

If BV is a predictor for adverse pregnancy outcome, screening during pregnancy and subsequent treatment might be rational. McGregor[9] has recommended screening all pregnant women and treating those with BV. He showed a significant reduction in preterm deliveries among women who were treated for BV during pregnancy compared with

*Correspondence to: Dr. Jane Boris, Department of Obstetrics and Gynecology, Hospital of Skaraborg Skövde, S-541 85 Skövde, Sweden.

Received 9 November 1997

Accepted 11 February 1998

women with BV who were not treated. However, whether pregnant women with asymptomatic BV should be treated remains controversial.[10, 11]

Recently, it has been shown that the presence of BV decreases spontaneously as the pregnancy proceeds. Bacterial vaginosis persists in fewer than 60% of women from the first to the third trimester of pregnancy. Women without BV very rarely develop BV during pregnancy.[12, 13]

The symptoms of BV are nonspecific and include troublesome vaginal discharge, primarily a foul-smelling discharge.[14, 15] How the symptoms may change during pregnancy in relation to BV is unknown, but in a discussion of the "screen and treat" method, this knowledge seems crucial. In an attempt to identify women with increased risk of BV, we conducted a study to investigate the symptomatology of BV and the longitudinal changes of BV during pregnancy.

### SUBJECTS AND METHODS

The study was carried out in a midwife clinic in the city of Aarhus, Denmark, and the protocol was approved by the regional ethics committee. Women scheduled for routine prenatal visits in the 30th and 33rd week of pregnancy were invited to participate.

Women below 18 years of age and women who had received antibiotics within two weeks prior to the visit were excluded. Women who entered the study during the 30th gestational week were asked to participate in a similar examination in the 33rd gestational week if possible.

Prior to vaginal examination, all women were interviewed about obstetric complications during previous pregnancies and the present pregnancy. They were asked about the presence of specific symptoms related to vaginal discharge, i.e., whether the discharge was increased, malodorous, troublesome, or caused itching.

The examination included evaluation of vaginal discharge with respect to amount, consistency, and color, pH-measurement of discharge from the posterior fornix with indicator paper, and an amine test (mixing two drops of 10% potassium hydroxide with discharge from the posterior fornix). A wet smear was made at each examination by adding one drop of physiological saline solution to discharge from the posterior fornix. The wet smear was examined for motile trichomonas and clue cells immediately using a phase contrast microscope with 400x magnification in 5–10 high-power fields. Clue cells were identified as vaginal epithelial cells with indistinct cell borders obscured by numbers of attached bacteria.[16] Bacterial vaginosis was then diagnosed according to the classical criteria established by Amsel et al.[14] with presence of three of the following four criteria: 1) thin and homogeneous vaginal discharge; 2) vaginal pH less than 4.5; 3) a positive amine test; and 4) the presence of clue cells identified in the wet smear.

**TABLE 1. Characteristics of women in the study and the background population of pregnant women who delivered at the Department of Obstetrics and Gynecology, Aarhus University Hospital, Aarhus, Denmark, during 1991**

| Maternal characteristics | Study participants n = 305 | | Nonparticipants n = 3,818 | |
|---|---|---|---|---|
| | Mean | (SD) | Mean | (SD) |
| Age (years) | 29.1 | (4.6) | 28.8 | (4.9) |
| Prepregnant weight (kg) | 61.4 | (10.4) | 61.1 | (10.0) |
| Height (cm) | 167.5 | (6.0) | 167.3 | (6.3) |
| Smokers (%) | 42.3 | | 45.0 | |
| Nullipara (%) | 54.8 | | 51.1 | |

All women were examined for *Chlamydia trachomatis, Neisseria gonorrhoeae,* and group B streptococci using standard laboratory tests. Women found to be positive for chlamydia were treated with oral erythromycin (500 mg twice a day for 10 days).

All interviews and vaginal examinations were carried out by the same person (JB).

All women in the study were also part of a large cohort study described in detail elsewhere.[17] This study provided information on medical and obstetric data and lifestyle factors.

Categorical variables were compared by chi-square test. A two-tailed $P$ value less than 0.05 was considered statistically significant.

### RESULTS

The study comprised 305 women. With respect to various background characteristics, the participants were similar to the entire population of pregnant women who gave birth at our department during 1991 (Table 1).

The prevalence of BV among women who entered the study group (women at 30–33 weeks gestation) was 16% (n = 49). Women with BV were significantly more often smokers than women with-

TABLE 2. Characteristics of women with and without bacterial vaginosis (BV) in early third trimester

| | BV n = 49 | | Non-BV n = 256 | |
|---|---|---|---|---|
| Maternal characteristics | Mean | SD | Mean | SD |
| Age (years) | 30 | (4.5) | 29 | (4.7) |
| Prepregnant weight (kg) | 62 | (10.3) | 61 | (10.4) |
| Height (cm) | 167 | (5.9) | 168 | (6.4) |
| Nullipara (%)[a] | 39 | | 46 | |
| Previous preterm delivery (%)[a] | 10 | | 8 | |
| Smoker (%)[b] | 52 | | 34 | |
| Employed (%)[a] | 51 | | 53 | |
| Single (%)[a] | 11 | | 7 | |
| Sexually active during third trimester (%)[a] | 65 | | 59 | |
| Urinary tract infection during pregnancy (%)[a] | 15 | | 9 | |
| Vaginal bleeding in early pregnancy (%)[a] | 10 | | 5 | |
| Premature contractions (%)[a] | 12 | | 17 | |

[a]Difference was not statistically significant.
[b]$P < 0.05$.

TABLE 3. Symptoms in early third trimester among 305 pregnant women with and without bacterial vaginosis (BV)[a]

| | BV (n = 49) | Non-BV (n = 256) |
|---|---|---|
| Symptoms | % | % |
| Increased discharge | 76 | 68 |
| Vaginal itching and burning | 16 | 15 |
| Malodorous discharge | 26 | 23 |
| Pronounced malodorous discharge | 4 | 5 |
| Troublesome discharge | 32 | 27 |

[a]Differences were not statistically significant.

out BV. However, women with and without BV were comparable with respect to other characteristics such as age, marital and employment status, parity, and complications during pregnancy (including urogenital infections, frequent and painful contractions, and vaginal bleeding) (Table 2). More than two thirds of all the women reported an increased amount of vaginal discharge, and when specifically asked, about one fourth had noticed a more malodorous discharge during pregnancy (Table 3). No discrepancy in the recordings of the discharge (the amount, smell, or whether it caused itching or burning sensation) was found between women with and without BV. The women with BV did not feel more worried about the change in character of their discharge than women without BV, and none of the participating women had seen a doctor because of the discharge changes.

Eight women in our study delivered preterm, making an overall rate of preterm delivery of 2.6% (8/305). The incidence of preterm delivery among women with BV was 4.0% (2/49), and among women without BV the incidence was 2.4% (6/256). This difference was not statistically significant.

The three-week follow-up examination of 125 of the 305 women initially examined revealed that 62% (13/21) of the women with BV at the first examination still had BV three weeks later, while 38% (8/21) no longer fulfilled the diagnostic criteria for BV at the second examination. Of these eight women without BV at the second examination, one did not fulfill any of Amsel's criteria for BV while the other seven had a raised pH or a positive amine test or both and four had a smear dominated by very short lactobacilli. Of the 104 women initially found to be without BV, none had developed BV by the second visit three weeks later.

A total of 5% of the women were infected with *Chlamydia trachomatis* when they entered the study in the 30th to 33rd week of gestation. Group B streptococci were cultured in 9%, and no women were found to have *Neisseria gonorrhoeae. Trichomonas vaginalis* was not found in any wet smear. No difference in the presence of any of these organisms were found between women with and without BV (Table 4).

### DISCUSSION

We found a prevalence of BV of 16% among women who attended routine antenatal care at 30 weeks gestation. This is in accordance with findings from another Danish study, in which researchers found a prevalence of BV of 14% among 2,927 pregnant women (personal communication with Poul Thorsen, MD). BV has been diagnosed in 12–33%[9, 13] of pregnant women in other populations. However, studies on BV in pregnancy are difficult to compare, mostly because of the heterogeneity among study populations, differences in gestational age at examination, and differences in the diagnostic criteria. Among 10,397 American pregnant women participating in the Vaginal Infection and Prematurity Study[3] the prevalence of BV was 16% among women at 23–26 weeks of gestation.

TABLE 4. Findings in early third trimester among 305 pregnant women with and without bacterial vaginosis (BV)[a]

| Findings | BV (n = 49) % | Non-BV (n = 256) % |
|---|---|---|
| *Chlamydia trachomatis* infection | 4 | 5 |
| Group B streptococci | 6 | 10 |
| *Neisseria gonorrhoeae* infection | 0 | 0 |
| *Trichomonas vaginalis* infection | 0 | 0 |

[a]Differences were not statistically significant.

The most prominent symptom of BV is probably malodorous discharge[15] and not increased amount of discharge. We presumed that by more specific and precise questioning about the characteristics of the vaginal discharge, we would be able to identify women with BV. However, more than two thirds of the women in our study reported increased amount of discharge and nearly one fourth reported malodorous discharge. No difference in the reporting of amount or odor of the discharge was observed between women with and without BV, and three of four of the women with BV did not report malodorous discharge at all, even when asked specifically. Thus, asymptomatic BV was more common among these pregnant women than symptomatic BV. Accordingly, the ability to predict which women have BV by careful questioning about specific symptoms does not seem to be possible.

None of the women in our study spontaneously complained about discharge, and only a very few felt worried by the character of the discharge when questioned. Surprisingly, about 25% of all the women without BV felt they had a foul-smelling discharge when asked specifically. One explanation might be that body secretions are a subject fraught with taboo and guilt, and that many women consider any smell from the vagina as a foul-smelling odor.

McGregor et al.[9] failed to reduce the overall incidence of adverse pregnancy outcome by treating only women with symptomatic BV. When all women were screened and women with BV were treated, the incidence of preterm delivery was reduced. This is supported by our findings, which indicate that screening for BV only among symptomatic women is insufficient when trying to predict women at increased risk of having BV.

Only a few studies deal with the spontaneous course of untreated BV during pregnancy.[12, 13] In our study, more than one third of the women with BV at 30 gestational weeks did not fulfill the criteria for BV three weeks later. None of the women without BV had BV at the follow-up visit.

The relative risk of preterm delivery for women with BV compared with women without BV has been reported to be between 1.4 and 6.9.[3, 18] We found that women with BV more often had a preterm delivery than women without BV, although the association was not significant (odds ratio was 1.8 [confidence interval was 0.4–9.0]). Recent studies indicate that the strongest association between BV and preterm delivery is seen when BV is diagnosed in early pregnancy.[7, 13] In our study women were included in early third trimester and for this reason our risk estimate may be underestimated. However, the small number of women in our study complicates the interpretation.

We observed a higher frequency of smokers among women with BV than among women without BV. This has also been noted by others.[13, 19, 20] In studies of abnormal vaginal flora and preterm delivery, smoking has usually been considered a potential confounder. However, whether the association between smoking and BV is causal or statistical is still controversial, and smoking might be an intermediate factor in the cause-effect relation. Thus, controlling for smoking when calculating the relative risk of preterm delivery among women with BV would result in a lowering of the risk estimates.

In summary, we showed that asymptomatic BV in pregnancy is common, and not even by asking women specifically about the character of their discharge could we identify women with BV. Women with BV are more often smokers than women without BV.

### ACKNOWLEDGMENTS

We are indebted to Jakob Hjort for preparing the data for analyses and P. G. Larsson for invaluable advice regarding the diagnostic process of BV.

### REFERENCES

1. Eschenbach DA, Gravett MG, Chen KC, Hoyme UB, Holmes KK: Bacterial vaginosis during pregnancy. An association with prematurity and postpartum complications. Scand J Urol Nephrol Suppl 86:213–222, 1984.
2. Martius J, Eschenbach DA: The role of bacterial vaginosis as a cause of amniotic fluid infection, chorioamnionitis and prematurity–a review. Arch Gynecol Obstet 247:1–13, 1990.
3. Hillier SL, Nugent RP, Eschenbach DA, et al.: Association between bacterial vaginosis and preterm delivery of a low-birth-weight infant. N Engl J Med 333:1737–1742, 1995.
4. McCoy MC, Katz VL, Kuller JA, Killam AP, Livengood CHI: Bacterial vaginosis in pregnancy: an approach for the 1990s. Obstet Gynecol Surv 50:482–488, 1995.
5. Watts DH, Krohn MA, Hillier SL, Eschenbach DA: Bacterial vaginosis as a risk factor for post-Cesarean endometritis. Obstet Gynecol 75:52–58, 1990.
6. Andrews WW, Goldenbarg RL, Hauth JC: Preterm labor: Emerging role of genital tract infections. Infect Agents Dis 4:196–211, 1995.
7. Riduan JM, Hillier SL, Utomo B, Wiknjosastro G, Linnan M, Kandun N: Bacterial vaginosis and prematurity in Indonesia: Association in early and late pregnancy. Am J Obstet Gynecol 169:175–178, 1993.
8. Hay PE, Lamont RF, Taylor-Robinson D, Morgan DJ, Ison C, Pearson J: Abnormal bacterial colonisation of the genital tract and subsequent preterm delivery and late miscarriage [see comments]. Br Med J 308:295–298, 1994.
9. McGregor JA, French JI, Parker R, et al.: Prevention of premature birth by screening and treatment for common genital tract infections: Results of a prospective controlled evaluation. Am J Obstet Gynecol 173:157–167, 1995.
10. Yoong A: Bacterial vaginosis and preterm delivery. Spurious link may lead to overtreatment [letter; comment]. Br Med J 308:787–788, 1994.
11. Priestley CJ, Nageswaran A, Dhar J: Bacterial vaginosis and preterm delivery. Little evidence of causal association [letter; comment]. Br Med J 308:788, 1994.
12. Platz-Christensen JJ, Pernevi P, Hagmar B, Andersson E, Brandberg A, Wiqvist N: A longitudinal follow-up of bacterial vaginosis during pregnancy. Acta Obstet Gynaecol Scand 72:99–102, 1993.
13. Hay PE, Morgan DJ, Ison CA, et al.: A longitudinal study of bacterial vaginosis during pregnancy. Br J Obstet Gynaecol 101:1048–1053, 1994.
14. Amsel R, Totten PA, Spiegel CA, Chen K, Eschenbach DA, Holmes KK: Nonspecific vaginitis. Diagnostic criteria and microbial and epidemiologic associations. Am J Med 74:14–22, 1983.
15. Eschenbach DA, Hillier S, Critchlow C, Stevens C, DeRouen T, Holmes KK: Diagnosis and clinical manifestations of bacterial vaginosis. Am J Obstet Gynecol 158:819–828, 1988.
16. Gardner HL, Dukes CD: *Haemophilus vaginalis* vaginitis. A newly defined specific infection previously classified as "nonspecific" vaginitis. Am J Obstet Gynecol 69:962–976, 1955.
17. Hedegaard M, Henriksen TB, Sabroe S, Secher NJ: Psychological distress in pregnancy and preterm delivery. Br Med J 307:234–239, 1993.
18. Kurki T, Sivonen A, Renkonen OV, Savia E, Ylikorkala O: Bacterial vaginosis in early pregnancy and pregnancy outcome. Obstet Gynecol 80:173–177, 1992.
19. Larsson PG, Platz-Christensen JJ, Sundstrüm E: Is bacterial vaginosis a sexually transmitted disease? Int J STD AIDS 2:362–364, 1991.
20. Llahi-Camp JM, Rai R, Ison C, Regan L, Taylor-Robinson D: Association of bacterial vaginosis with a history of second trimester miscarriage. Hum Reprod 11:1575–1578, 1996.
